# Accelerated Fast Spin-Echo Magnetic Resonance Imaging of the Heart Using a Self-Calibrated Split-Echo Approach

**DOI:** 10.1371/journal.pone.0094654

**Published:** 2014-04-11

**Authors:** Sabrina Klix, Fabian Hezel, Katharina Fuchs, Jan Ruff, Matthias A. Dieringer, Thoralf Niendorf

**Affiliations:** 1 Berlin Ultrahigh Field Facility (B.U.F.F.), Max-Delbrueck-Center for Molecular Medicine, Berlin, Germany; 2 Siemens Healthcare, Erlangen, Germany; 3 Experimental and Clinical Research Center (ECRC), a joint cooperation between the Charité Medical Faculty and the Max-Delbrueck-Center for Molecular Medicine, Berlin, Germany; National Taiwan University, Taiwan

## Abstract

**Purpose:**

Design, validation and application of an accelerated fast spin-echo (FSE) variant that uses a split-echo approach for self-calibrated parallel imaging.

**Methods:**

For self-calibrated, split-echo FSE (SCSE-FSE), extra displacement gradients were incorporated into FSE to decompose odd and even echo groups which were independently phase encoded to derive coil sensitivity maps, and to generate undersampled data (reduction factor up to R = 3). Reference and undersampled data were acquired simultaneously. SENSE reconstruction was employed.

**Results:**

The feasibility of SCSE-FSE was demonstrated in phantom studies. Point spread function performance of SCSE-FSE was found to be competitive with traditional FSE variants. The immunity of SCSE-FSE for motion induced mis-registration between reference and undersampled data was shown using a dynamic left ventricular model and cardiac imaging. The applicability of black blood prepared SCSE-FSE for cardiac imaging was demonstrated in healthy volunteers including accelerated multi-slice per breath-hold imaging and accelerated high spatial resolution imaging.

**Conclusion:**

SCSE-FSE obviates the need of external reference scans for SENSE reconstructed parallel imaging with FSE. SCSE-FSE reduces the risk for mis-registration between reference scans and accelerated acquisitions. SCSE-FSE is feasible for imaging of the heart and of large cardiac vessels but also meets the needs of brain, abdominal and liver imaging.

## Introduction

In current clinical cardiac MR (CMR) inversion recovery prepared black blood fast spin-echo (FSE) techniques [1] are commonly used for anatomical and morphological imaging of the heart and large vessels [1], [2]. Clinical applications also include probing for myocardial edema, assessment of amyloidosis and myocardial tissue characterization using parametric mapping [3]–[8]. Black blood FSE imaging of the heart can be time consuming since it is commonly confined to a single slice per breath-hold due to the competing constraints of spatial resolution and physiological motion which dictate the viable window of data acquisition.

Acceleration through parallel imaging seeks to relax the speed constraints of FSE by using radiofrequency (RF) coil arrays in conjunction with k-space domain techniques [9] - exemplified by the original SMASH (SiMultaneous Acquisition of Spatial Harmonic) and GRAPPA (GeneRalized Autocalibrating Partially Parallel Acquisitions) algorithms [10], [11] - or image domain techniques - represented by the original Cartesian SENSE (SENSitivity Encoding) formulation [12] - for unfolding of aliased voxels that result from undersampling. One practical implication is that calibration of the component coil sensitivity profiles is required. Separate calibration using external reference scans prior to accelerated imaging has been used successfully for breath-hold and free breathing CMR [13]–[18] including black blood FSE [19], though the presence of cardiac and respiratory motion can present challenges for external sensitivity calibration [20], [21].

The potential for motion and hence mis-registration between calibration scans and accelerated scans have prompted the development of self-calibrating parallel imaging approaches, which have seen extensive use in CMR [11], [22]–[24]. Self-calibration is conveniently incorporated in the time-domain approaches. Self-calibrated SENSE has been demonstrated for accelerated spatio-temporal hybrid techniques which rely on dynamic data [25]–[31]; a requirement which is not met by standardized CMR protocols used for single cardiac phase black blood FSE imaging of the heart [32], [33].

Recognizing the needs of cardiac MRI and the opportunities of black blood FSE imaging it is conceptually appealing to pursue accelerated, self-calibrated FSE techniques. For this reason this work proposes a modified FSE variant which uses a split-echo approach [34], [35]. For this purpose the full echo of coherent FSE is decomposed into two parities, which can be independently phase encoded (i) using regular sampling to derive coil sensitivity profiles and (ii) k-space undersampling to accelerate acquisitions. Consequently, reference and undersampled data are acquired simultaneously which makes an external reference scan obsolete. The proposed FSE variant is referred to self-calibrated, split-echo FSE (SCSE-FSE) for reasons of brevity. The feasibility of SCSE-FSE is carefully examined in phantom studies. The immunity of SCSE-FSE for motion induced mis-registration between reference and undersampled data is demonstrated in phantom studies using a dynamic MR compatible left ventricular model [36]. For comparison, traditional FSE variants [34], [37], [38] are applied. The applicability of black blood prepared SCSE-FSE for cardiac MR is assessed in healthy volunteers and benchmarked versus traditional FSE. These efforts include accelerated multi-slice per breath-hold imaging and examination of motion induced mis-registration. The merits and limitations of SCSE-FSE are discussed and implications for clinical cardiac MR are considered.

## Materials and Methods

### MR methodology

The underlying principle of Rapid Acquisition with Refocusing Echoes/Fast Spin Echo (RARE/FSE) is the acquisition of an echo train generated by an initial excitation pulse and a train of *n* equidistant refocusing pulses (α) whereby each echo is independently phase encoded [39], [40]. Nutation angles of nominal α≠180° induce a number of coherence pathways giving rise to single odd/even echo groups [37]. The two echo groups superimpose coherently provided that (i) a perfect trimming of the frequency encoding gradient is achieved and (ii) the Carr-Purcell-Meiboom-Gill (CPMG) condition [41] is satisfied (coherent FSE, [37]). To eliminate interferences between odd and even echo groups one echo group can be shifted out of the acquisition window using an additional crusher gradient along the frequency encoding direction, which is designated as displaced FSE [37]. Consequently one echo group does not contribute to the signal.

In split-echo FSE the echo groups are separated by a mis-trimmed frequency encoding gradient [34], [35], [42]. This can be realized by crusher gradients situated about the acquisition epoch and/or by an imbalanced read dephasing gradient. In split-echo FSE, odd and even echo groups experience the same phase encoding and are simultaneously recorded within the acquisition period.

**Figure 1:**
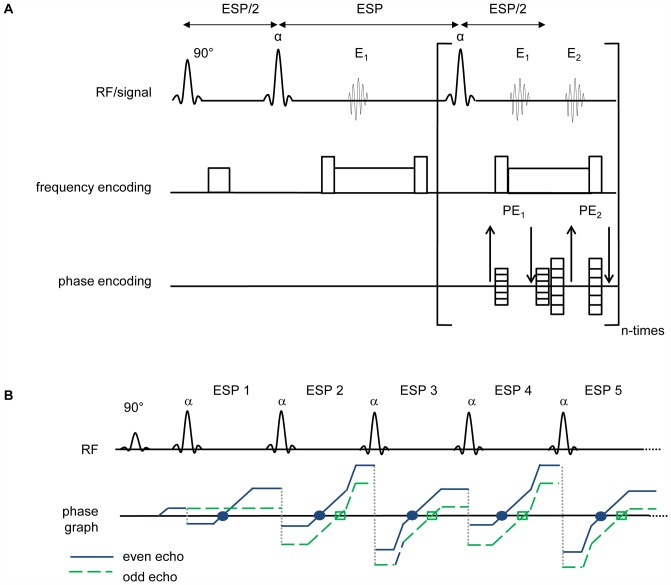
Basic scheme of the self-calibrated, split-echo FSE (SCSE-FSE) technique. **A**) Basic scheme for the first three RF pulses. Unlike coherent FSE odd (E_1_) and even (E_2_) echo groups are separated from each other in SCSE-FSE. Both echo groups are phase encoded (PE_1_, PE_2_) independently. One echo group is used to generate the coil sensitivity map (E_1_). The other echo group is employed to generate undersampled data which are unfolded using SENSE reconstruction (E_2_). **B**) Extended phase graph together with the position of the PF pulses to illustrate the evolution of the magnetization in SCSE-FSE for the first five RF pulses.

For self-calibrated, split-echo FSE independent phase encoding schemes PE_1_ and PE_2_ are applied for even and odd echo parities as illustrated in Figure 1A. One echo group is phase encoded to form a fully sampled reference data set for determination of B_1_ coil sensitive profiles (E_1_) as outlined in Figure 1A. The other echo group is phase encoded to generate an accelerated data set with an undersampling factor of R (E2). This can be achieved by dividing the moment of the phase encoding gradient used for the reference data by R. For this purpose a single net phase encoding gradient with a duration t_grad_<300 µs was used in our proof-of-principle implementation. With this phase encoding scheme odd/even echoes can serve as reference/undersampled data and vice versa with a spatial resolution of the reference map of 1/R of the final image. Figure 1B demonstrates the magnetization pathways based on the extended phase graph algorithm [43]–[45] for both echo groups for the first five echo spacings (ESP).

### Image Acquisition and Reconstruction

Image acquisition and reconstruction involved SCSE-FSE and the conventional FSE variants (coherent FSE, split-echo FSE and displaced FSE) including regular density and undersampled phase encoding schemes. Imaging parameters were kept identical for all FSE variants as outlined in detail in the phantom experiments and cardiac imaging section.

Raw data were exported from the scanner and reconstructed offline using an in-house implementation in MATLAB (MATLAB, The MathWorks, Inc., Natwick, USA) and the PULSAR toolkit [46] for GRAPPA [11] and SENSE [12] reconstruction.

The following strategies were performed to reconstruct the FSE variants:

- **fully sampled coherent FSE and displaced FSE**
  ○ magnitude images were derived from element-wise 2D FFT reconstruction from each coil followed by sum of squares (SOS) combination [47].
- **fully sampled split-echo FSE**
  ○ two magnitude images were obtained after extraction of odd and even echo groups from the raw data followed by 2D-FFT and SOS. The two images were added up to form a single image.
- **fully sampled SCSE-FSE,**
  ○ odd echoes were used to generate the final image by using 2D FFT and SOS.
- **undersampled coherent FSE,**
  ○ displaced-FSE and split-echo FSE data were reconstructed using a separate reference scan with 32 lines together with SENSE reconstruction.
  For comparison, 31 internal reference lines were used for self-calibrated GRAPPA reconstruction.
- **undersampled SCSE-FSE,**
  ○ data were reconstructed using even echoes for coil calibration and undersampled data derived from odd echoes together with SENSE reconstruction.

### MR-Hardware

Imaging was conducted using a wide-bore 3.0 T MR system (Magnetom Verio, Siemens Healthcare, Erlangen, Germany). A body RF coil was applied for signal transmission. For reception a 32 channel head coil (Siemens Healthcare, Erlangen) and a 32 channel cardiac coil array (IN VIVO Corp., Gainsville, USA) were applied. An MR stethoscope (easyACT, MRI.TOOLS GmbH, Berlin, Germany) was used for cardiac triggering [48]–[50].

### Phantom experiments

Phantom experiments using conventional FSE variants and SCSE-FSE were performed using (i) a stationary object and (ii) a dynamic model of the left ventricle [36]. For the stationary object a spherical phantom (inner diameter  = 15 cm, D165, Siemens Healthcare, Erlangen, Germany) was used. The phantom is made of Plexiglas and filled with oil. Imaging parameters were set to: Matrix size 512×256, echo train length  = 16, number of dummy echoes  = 8 [51], in-plane spatial resolution  =  (0.9×0.9) mm^2^, slice thickness  = 5 mm, effective echo time TE_eff_  = 61 ms, repetition time TR  = 1000 ms, receiver bandwidth  = 454 Hz/pixel, nominal refocusing pulse  = 180°. For parallel imaging acceleration factors of up to R = 3 were applied for all FSE variants.

For the dynamic phantom an MR compatible left ventricle model was employed [36]. For this purpose an artificial ventricle was formed of silicon based on the geometric approximation of a paraboloid. The ventricle model exhibits T_1_ and T_2_ relaxation times that mimic that of the myocardium. A pump system supplied the ventricle with pulsatile flow of a water/glycerol mixture that exhibited the viscosity of human blood. The pump inflated and deflated the ventricle to mimic ventricular contraction. Cardiac triggering was used. Imaging parameters were identical to those used for the static phantom with the exception of: in-plane spatial resolution  =  (1.3×1.3) mm^2^, slice thickness  = 8 mm, TE_eff_  = 77 ms, receiver bandwidth  = 454 Hz/pixel.

To examine the propensity for mis-registration between reference data and undersampled data the ventricle model was moved by 15 mm along the long axis of the phantom after the acquisition of the reference data.

### Ethics Statement

For the *in vivo* feasibility study, eight healthy subjects without known history of cardiac diseases (mean age: 293 years, 6 men, 2 women, mean BMI: 242.3 kg/m^2^, mean heart rate: 7515 bpm) were included after due approval by the local ethical committee (registration number EA1/151/10 Ethikkomission Charité-Universitätsmedizin, Berlin, Germany). Informed written consent was obtained from each volunteer prior to the study.

### Cardiac imaging

For cardiac imaging slice positioning was carried out following international consensus. For this purpose the heart was localized in three orthogonal thoracic slices in each spatial orientation using low-resolution SSFP scout images. The long axis of the left ventricle was dissected twice, and finally a stack of short axes was obtained. These slices provided the basis for planning the standard long axis views (four-chamber, three-chamber and two-chamber view) derived from 2D CINE SSFP imaging (in-plane resolution  =  (1.7×1.7) mm^2^, slice thickness  = 6 mm, TE  = 1.3 ms, TR  = 2.7 ms, matrix size  = 192×192, nominal flip angle  = 45°). Based on the four-chamber view (4CV), a mid-ventricular short axes view (SAX) parallel to the mitral valve was planned.

For black blood imaging, double inversion recovery prepared coherent FSE, displaced FSE, split-echo FSE and SCSE-FSE were conducted. For all FSE variants imaging parameters were set to: in-plane resolution  =  (1.2×1.2) mm^2^, slice thickness  = 5 mm, TE_eff_  = 54 ms, TR  = 1 R-R interval, matrix size  = 512×256, receiver bandwidth  = 454 Hz/pixel, ESP  = 4.9 ms.

Two strategies were used to examine artifacts due to mis-registration between reference data and undersampled data in cardiac imaging:

- The reference map was shifted 5 or 10 pixels parallel to the long axis of the heart to mimic physiological motion induced mis-registration.
- Reference data were acquired during systole and undersampled image data were acquired during mid-diastole.

### Image Quality Assessment

For image quality assessment, signal-to-noise ratio (SNR) and g-factor maps were calculated using an acceleration factor of up to R = 4. SNR maps were derived from a time series of images using SNR  =  x_t_(r)/σ_t_(r) with x_t_(r) being the mean signal intensity of pixel r over time t and σ_t_ (r) being the standard deviation (SD) of the signal intensity in pixel r over time t for 16 images. For closer examination mean SNR values and standard deviation are reported for a ROI (diameter  = 6.6 cm) placed in the center of an axial slice of the phantom. G-factor maps were extracted during SENSE reconstruction.

For point spread function (PSF) analysis, static phantom measurements were conducted with phase encoding gradients turned off. The full-width-half-maximum (FWHM) was calculated as a measure for the quality of the PSF.

## Results

### Phantom Experiments

**Figure 2:**
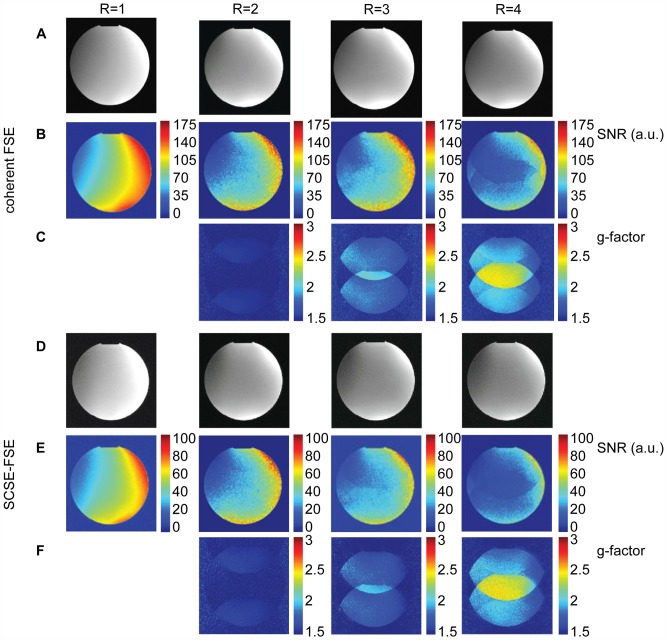
SNR maps and g-factor maps for coherent FSE and SCSE-FSE. **Top**) Synopsis of results derived from coherent FSE imaging of a phantom R = 1 (first column) together with SENSE reconstruction and reduction factors of R = 2 (second column), R = 3 (third column) and R = 4 (last column). SENSE reconstructed images (A); images scaled in SNR units (B) and g-factor maps (C) derived from SNR maps. SNR was found to be SNR  =  (63±7) for R = 2, SNR  =  (56±6) for R = 3 and SNR  =  (38±5) for R = 4. **Bottom**) Synopsis of results derived from SCSE-FSE imaging of a phantom R = 1 (first column) together with SENSE reconstruction and reduction factors of R = 2 (second column), R = 3 (third column) and R = 4 (last column). SENSE reconstructed images (D); images scaled in SNR units (E); g-factor maps (F) derived from SNR maps. Signal-to-noise ratio was SNR  =  (29±6), SNR  =  (26±3) and SNR  =  (21±3) for R = 2, R = 3 and R = 4.

T_2_ weighted imaging using all FSE variants and the static phantom was performed successfully. For regular k-space density sampling coherent FSE and split-echo FSE yielded SNR  = 107±10 for a ROI (D = 6.6 cm) placed in the center of an axial slice of the phantom. For the same ROI, displaced FSE and SCSE-FSE provide SNR  = 49±11 which is half of coherent FSE since only one echo group is used for the generation of the final image. For SCSE-FSE the extra phase encoding gradients PE_1_ and PE_2_ did not diminish image quality compared to coherent FSE as demonstrated in Figure 2A and 2D.

SNR maps and g-factor maps derived from accelerated coherent FSE and SCSE-FSE imaging are shown in Figure 2 using an acceleration factor up to R = 4. For a ROI placed in the center of an axial slice through the phantom coherent FSE yielded SNR  =  (63±7), SNR  =  (56±6) and SNR  =  (38±5) for R = 2, R = 3 and R = 4. In comparison, SCSE-FSE provided SNR  =  (29±6), SNR  =  (26±3) and SNR  =  (21±3) for R = 2, R = 3 and R = 4. SCSE-FSE revealed noise amplification which matches that of parallel imaging with conventional FSE.

**Figure 3:**
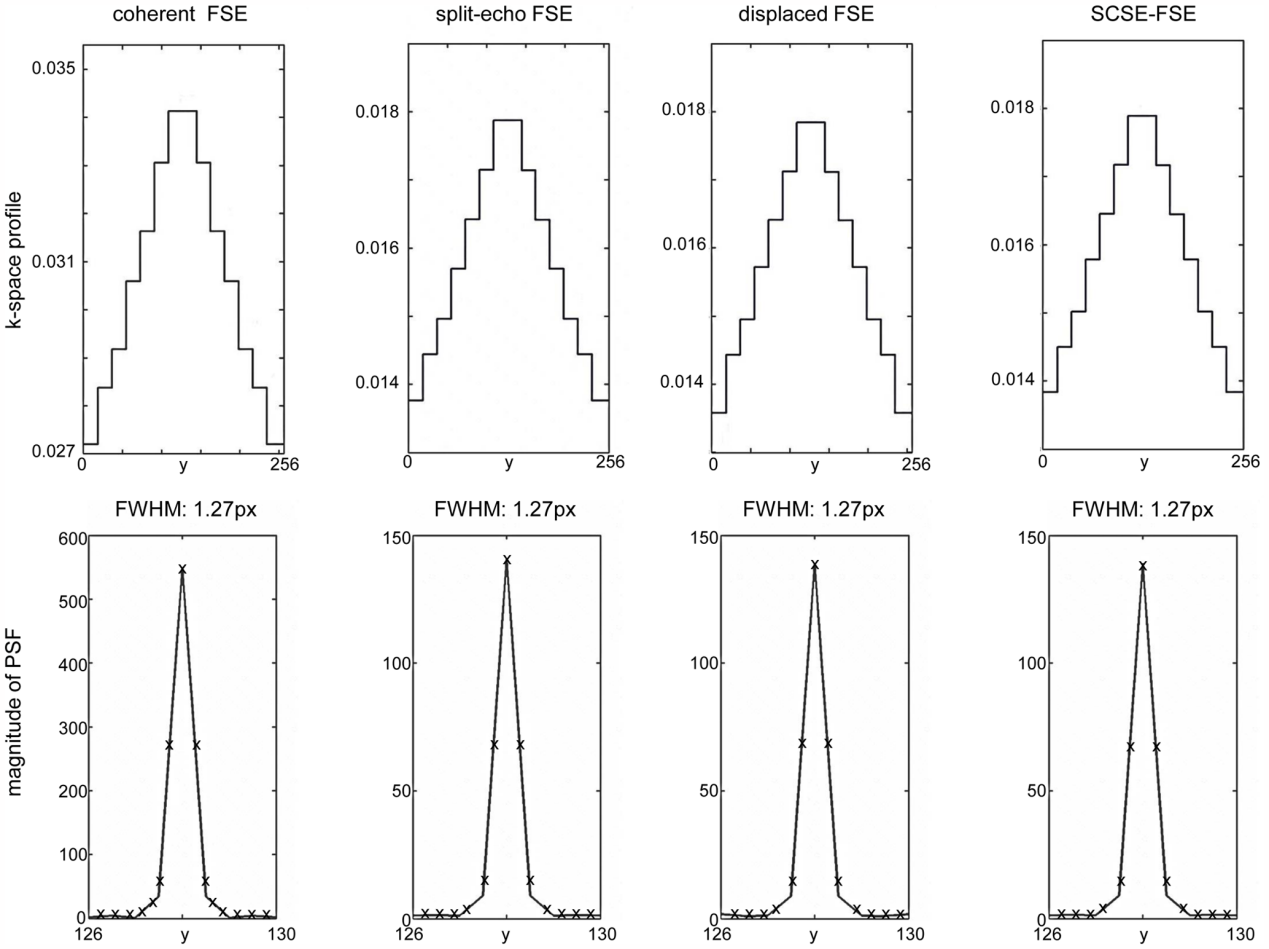
Point spread function (PSF) for coherent, split-echo, displaced and SCSE-FSE variants. The upper row depicts the k-space profile for each approach, reflecting the signal intensity of every k_y_-line via its integral. The FFT of this profile results in the point spread function; its magnitude is shown in the lower row. No major differences in the PSF were found for SCSE-FSE versus traditional FSE variants including coherent FSE, split-echo FSE, displaced FSE.

Figure 3 provides a synopsis of the results observed for PSF analysis. The upper row depicts the k-space profile for each approach, reflecting the signal intensity of every k_y_-line via its integral. The FFT of this profile results in the point spread function; its magnitude is shown in the lower row. No major differences in the point spread functions FWHM were found for the FSE variants used.

**Figure 4:**
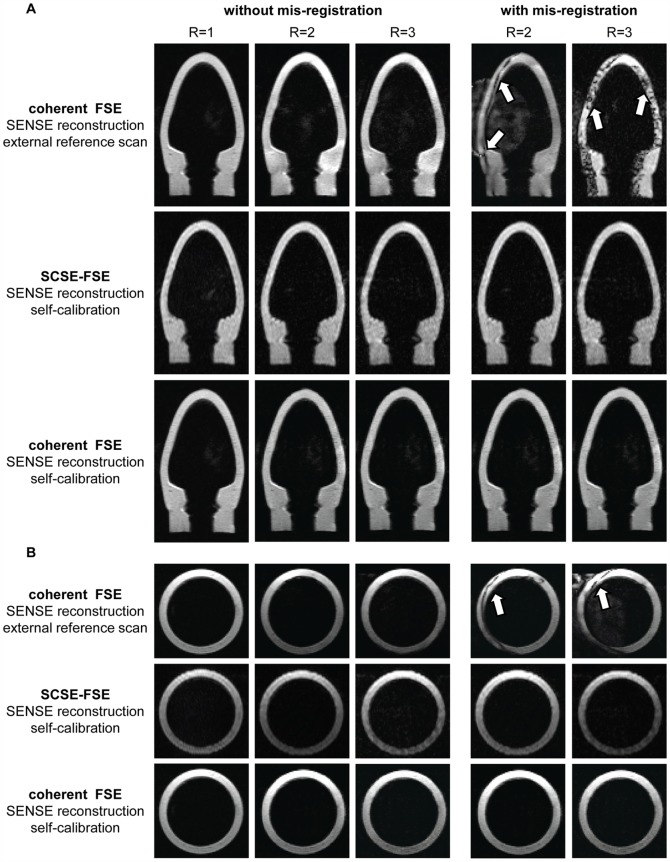
Long and short axis views of a dynamic left ventricle model derived from coherent FSE and SCSE-FSE acquisitions. **A**) Four chamber long axis views **B**) short axis views of a dynamic left ventricle model. A 15 mm movement of the phantom along the long axis of the left ventricle model was used to mimic bulk cardiac motion. The mis-registration between the external reference scans and the accelerated data derived from coherent FSE induced severe artifacts in the SENSE reconstructed images for R = 2 **(top)**. The artifacts were pronounced for R = 3. In comparison, SCSE-FSE was immune to bulk motion induced shifts in the phantom **(middle)** as was self-calibrated coherent FSE in conjunction with SENSE reconstruction **(bottom)**. For the latter coil sensitivity maps were deduced from 32 central k-space lines while a decimation factor of R = 2 and R = 3 was employed to generate undersampled data. Imaging parameters were: in-plane spatial resolution  =  (1.3×1.3) mm^2^, slice thickness  = 8 mm, TE_eff_  = 77 ms, receiver bandwidth  = 454 Hz/pixel for all data sets.

The propensity of coherent FSE and SCSE-FSE for bulk motion induced mis-registration between reference and undersampled data was examined using a dynamic model mimicking a cardiac left ventricle. Figure 4 illustrates the results obtained for long axis and short axis views of the dynamic phantom using coherent FSE together with an external reference scan. For a 15 mm mismatch in position along the long axis of the phantom between B_1_ calibration and accelerated imaging significant mis-registration artifacts were observed for coherent FSE. These artifacts were already present for two-fold accelerations and further pronounced for R = 3 for SENSE reconstruction as demonstrated in Figure 4.

In comparison, self-calibrated parallel imaging with SCSE-FSE using R = 2 and R = 3 was found to be immune to the 15 mm displacement as was self-calibrated coherent FSE using SENSE reconstruction.

### Cardiac imaging

**Figure 5:**
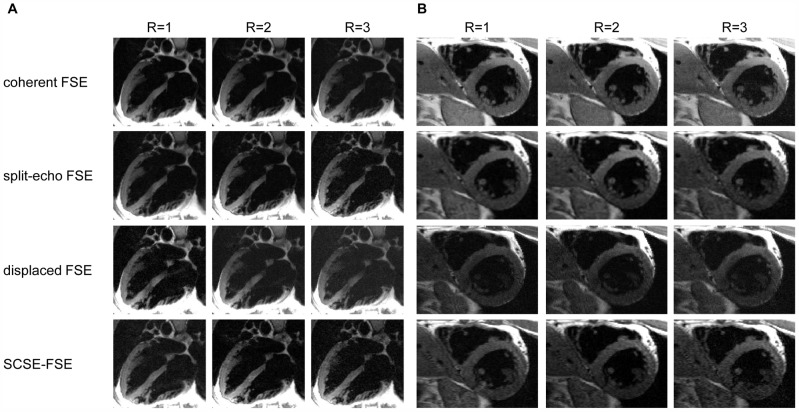
Four chamber long axis views and short axis views of the heart obtained with all FSE variants. **A**) Four chamber long axis views **B**) short axis views of the heart derived from double inversion recovery prepared acquisitions using coherent, split-echo, displaced and SCSE-FSE in conjunction with R = 1 **(left)**, R = 2 **(middle)** and R = 3 **(right)**. Imaging parameters were: in-plane resolution  =  (1.2×1.2) mm^2^ slice thickness  = 5 mm, TE_eff_  = 54 ms, matrix size  = 512×256, echo spacing 4.9 ms, receiver bandwidth  = 454 Hz/pixel for all data sets.

Four chamber long axis views and short axis views of the heart obtained with all FSE variants are shown in Figure 5. For parallel imaging acceleration factors of up to R = 3 were applied. The results indicate that SCSE-FSE imaging exhibits contrast properties which match that of the traditional FSE variants. Figure 5 demonstrates that SNR reduction intrinsic to SCSE-FSE versus coherent and split-echo FSE does not present a challenge for black blood imaging protocols given by the clinical guidelines [32].

**Figure 6:**
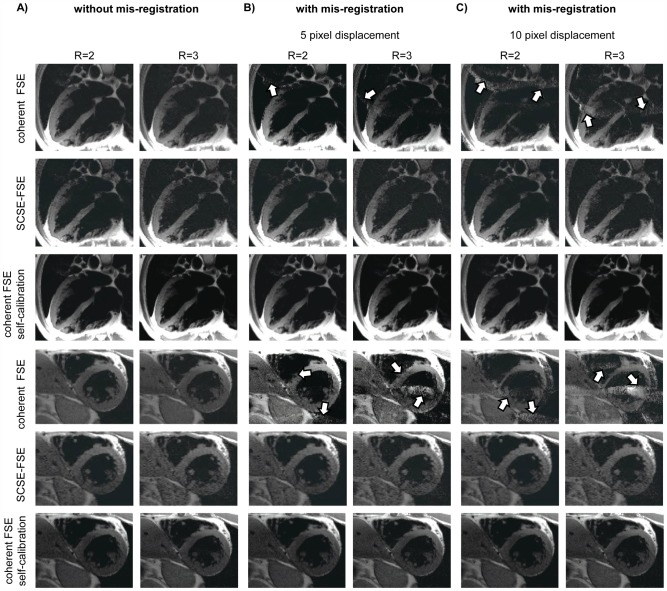
Illustration of mis-registration between reference data and undersampled data. The propensity to cardiac motion induced mis-registration between reference and undersampled data is demonstrated for four chamber (top) and short axis- views (bottom) of the heart derived from double inversion recovery prepared acquisitions using accelerated (R = 2, R = 3) coherent FSE and SCSE-FSE. **A**) Without mis-registration between reference data and undersampled image data. The images obtained for coherent FSE and SCSE-FSE compare well. **B**) With mis-registration by shifting the reference data 5 pixel parallel to the long axis of the heart, **C**) by shifting the reference data 10 pixel parallel to the long axis of the heart. Accelerated imaging (R = 2 and R = 3) with self-calibrated FSE was free of parallel imaging artifacts while aliasing artifacts were obtained for coherent FSE (white arrows). SENSE reconstructed, self-calibrated coherent FSE was immune to bulk motion induced shifts.

The immunity of SCSE-FSE for cardiac motion induced mis-registration between reference data and undersampled data is illustrated in Figure 6. For this purpose, the reference map was shifted 5 or 10 pixels parallel to the long axis of the heart to mimic a physiological motion induced mis-registration. For this setup, accelerated coherent FSE yielded severe artifacts for SENSE reconstruction using an external reference scan. In comparison, no image artifacts were observed for SCSE-FSE using self-calibration and SENSE reconstruction. SENSE reconstructed, self-calibrated coherent FSE was immune to bulk motion induced shifts.

**Figure 7:**
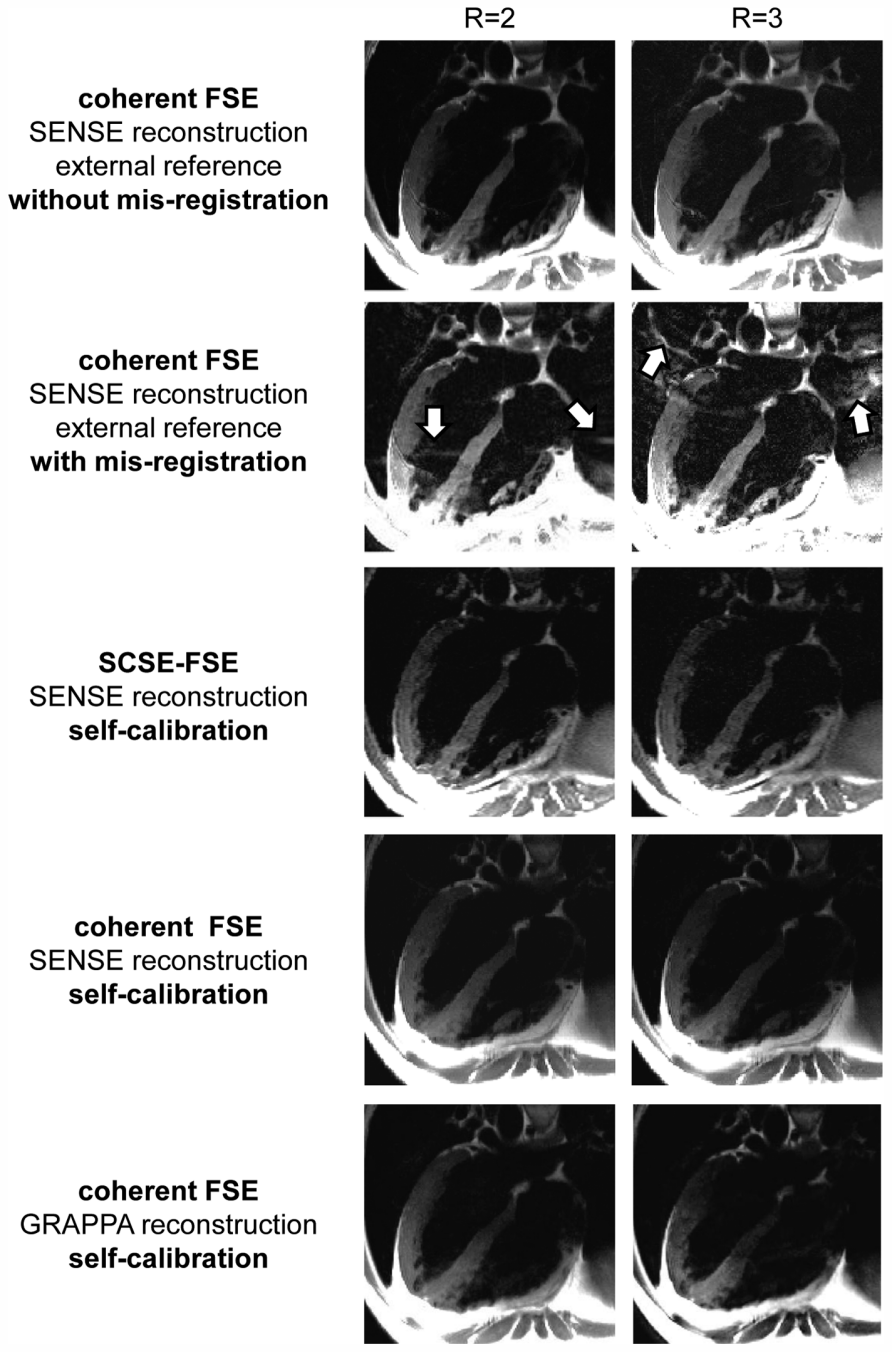
Illustration of mis-registration effects due to data acquisition during different cardiac phases. Four chamber long axis views derived from double inversion recovery prepared acquisitions using coherent FSE and SCSE-FSE (R = 2–3) in conjunction with SENSE and GRAPPA reconstruction. For the “*without mis-registration*” mode reference and accelerated data were acquired during the same cardiac phase (diastole). For the “*with mis-registration*” mode external reference data were acquired during systole for SENSE reconstruction while internal reference data used for self-calibration and undersampled data were recorded during diastole. For “self-calibration” reference and accelerated data were acquired during the same cardiac phase (diastole).

For further examination of mis-registration artifacts, external reference data were acquired in systole and undersampled image data were recorded during diastole. Figure 7 demonstrates mis-registration induced artifacts for coherent FSE in conjunction with SENSE reconstruction using an external reference scan for B_1_ calibration acquired during systole while accelerated data were acquired during diastole. Self-calibration together with GRAPPA reconstruction of coherent FSE and SCFSE-FSE in conjunction with SENSE reconstruction provided images free of artifacts since the internal reference data were acquired during the same cardiac cycle than the accelerated data.

**Figure 8:**
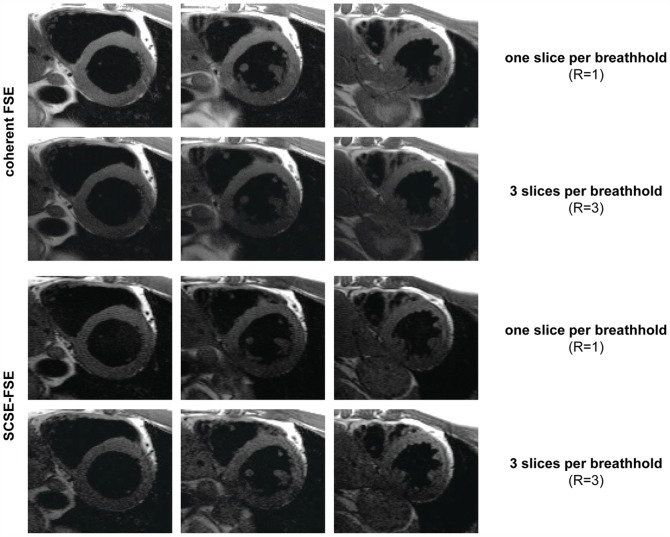
Transfer of enhanced imaging speed into improved heart coverage per breath-hold. Three short axis views of the heart derived from single breath-hold per slice acquisitions using coherent FSE and SCSE-FSE are shown. For comparison the same three short axis views of the heart were derived from three-fold accelerated single breath-hold acquisitions using coherent FSE and SCSE-FSE. The latter offers an effective examination time advantage of a factor of approximately six (assuming a 15 sec recovery after a breath-hold) over the single slice per breath-hold approach.

The speed gain of SCSE-FSE is demonstrated in Figure 8 using short axis views of the heart. For this purpose three slices were first derived from single breath-hold per slice acquisitions using coherent FSE and SCSE-FSE. For comparison the same three slices were derived from three-fold accelerated single breath-hold acquisitions using (i) coherent FSE in conjunction with SENSE reconstruction and (ii) SCSE-FSE. This approach resulted in an effective examination time advantage of a factor of approximately six (assuming a 15 sec recovery after a breath-hold) over fully sampled single breath-hold per slice FSE acquisitions.

## Discussion

This work demonstrates the feasibility of self-calibrated, split-echo FSE imaging in a stationary phantom, a dynamic cardiac left ventricle model, and in healthy volunteers. SCSE-FSE obviates the need of external reference scans for SENSE reconstructed parallel imaging with FSE. The application of independent phase encoding for both echo parities affords the simultaneous acquisition of (i) reference data used for B_1_ calibration and (ii) undersampled data. The self-calibrated approach reduces -the risk for mis-registration between external reference scans and accelerated acquisitions which frequently occurs in the presence of body or physiological motion when using external reference scans.

In today's clinical CMR practice self-calibration is conveniently incorporated in black blood FSE using k-space domain approaches for reconstruction. Clinical implementations of self-calibrated black blood FSE often come with no sacrifice in scan time for the acquisition of calibration signals and with no sacrifice for net acceleration. The scan time needed for the regular density acquisition of low frequency k-space calibration lines is however commonly compensated by increasing the undersampling rate for higher frequency k-space lines. This approach comes at the cost of increased noise amplification which is inherent to parallel imaging. This challenge is pronounced for cardiac FSE imaging where it is common to use a small matrix size along the phase encoding direction. This shifts the scan time weight to the number of calibration lines, in particular if half-Fourier or inner volume FSE techniques adapted to the size of the left ventricle or adjusted to the geometry of large vessels are applied. Our work adds to the literature by demonstrating that self-calibration can be conveniently incorporated into SCSE-FSE together with image domain SENSE reconstruction with no sacrifice in scan time and no compromise in the net reduction factor. Unlike the conventional approach the proposed SCSE-FSE approach does not exhibit the net-acceleration drawback of the conventional approach. SCSE-FSE does not use an increase in the undersampling rate for higher frequency k-space lines to compensate for the net acceleration penalty of conventional self-calibration techniques and hence does not suffer from the extra noise amplification induced by the higher undersampling rate for higher frequency k-space lines.

SCSE-FSE does not show additional noise amplification versus parallel imaging with conventional FSE. The basic concept using two independently echo groups of SCSE-FSE can be also applied to k-space domain reconstruction algorithms.

SCSE-FSE helps to address some of the limitations of previous self-calibrated approaches using SENSE reconstruction techniques. These approaches are commonly based upon spatio-temporal hybrid algorithms - a concept behind techniques such as UNFOLD-SENSE, TSENSE, auto-SENSE, and *k-t* SENSE - which are applied on a frame-by-frame basis [25]–[31]. These techniques share the need of a time series of data (for example CINE imaging or first pass bolus perfusion imaging) and hence do not support single cardiac phase black blood FSE imaging of the heart *per se*.

The speed gain offered by SCSE-FSE promises to extend the capabilities of black blood imaging of the heart from a single slice to multiple slices per breath-hold acquisitions. One practical implication is that SCSE-FSE would help to reduce examination times while improving both operator convenience and patient comfort. This is not limited to imaging anatomy and morphology of the heart and the large vessels but can be also put to good use for myocardial tissue characterization and probing of myocardial microstructure using apparent diffusion coefficient (ADC), T_2_ or T_2_^*^ mapping [3] or susceptibility weighted imaging and quantitative susceptibility mapping of the heart [52]. Susceptibility sensitization can be achieved in FSE by inserting an extra delay τ between the initial 90° excitation pulse and the first refocusing pulse [53]. Susceptibility sensitization introduces unknown phase shifts, which would lead to destructive interference between odd and even echo groups in case of coherent FSE and hence renders it unsuitable for T_2_^*^ weighted imaging/mapping. SCSE-FSE runs the advantage that odd and even echo groups are separated from each other, which helps to overcome the incompatibility of susceptibility weighted preparation experiments and conventional coherent FSE. SCSE-FSE provides also means for accelerating T_2_ mapping using multi-echo FSE techniques [54] or for dual contrast FSE [55].

The availability of multiple frames in a series of diffusion, T_2_ or T_2_^*^-weighted FSE images affords the opportunity to vary acquisition trajectories from image to image and to perhaps use spatio-temporal correlations measured from FSE training data [56], [57] to reassemble image components that are distributed in time and space. To this end, SCSE-FSE offers the simultaneous acquisition of training data and undersampled data without a scan time penalty. Provided that phase encoding is applied properly and that the same readout size is used for training and undersampled data, the training data can be even incorporated into the k-space of the undersampled data prior to reconstruction to preserve or enhance SNR, contrast and temporal fidelity [58], [59].

It is a recognized limitation of the proposed SCSE-FSE approach that the separation of odd and even echoes together with the use of only one echo group for image reconstruction comes with a SNR penalty of factor 2 versus coherent FSE, where both echo groups contribute to the signal. This caveat can be relaxed by using many element coil arrays tailored for cardiac MR [60]–[64] or by moving to CMR at magnetic field strengths of B_0_≥3.0 T [64]–[69].

The literature shows that the displacement of the epix or of the coronary arteries attached to the myocardium is about 2–4 cm across the cardiac cycle (diastole versus systole). The movement of the diaphragm that shifts the heart up and down is in between 3–5 cm across a respiratory cycle. Taking these physiological motions into account we opted for a close-to-reality scenario for the phantom experiments and used a 15 mm movement to demonstrate the effect of mis-registration. For the in vivo data a 5 and 10 pixel (which corresponds to approximately 5 mm and 10 mm) displacement was chosen with the corresponding results being shown in Figure 6. When using a smaller mismatch the displacement artifacts are shifted parallel to the long axis of the heart, but are still visible. Aliasing artifacts are more pronounced with a higher mismatch.

This study was designed to examine the feasibility and applicability of self-calibrated SCSE-FSE. Hence we decided not to apply any filter to the reference maps used for SENSE reconstruction and worked with the virgin raw data. Extra processing on the coil sensitivity maps might reduce artifacts caused by the mismatch between external reference scans and accelerated scans.

Furthermore, 3D volumetric acquisitions would serve to recover SNR via noise averaging [70], [71]. To this end, 3D volumetric acquisitions can benefit most from two-dimensional parallel imaging [16], [70], which would be supported by the proposed SCSE-FSE approach. SCSE-FSE is not limited to Cartesian phase encoding but is also compatible with non-cartesian FSE k-space trajectory variants [72]. SCSE-FSE supports double inversion recovery and triple inversion recovery preparation modules. To generalize, the initial excitation pulse can be substituted by any spin preparation that provides transverse magnetization. The self-calibrated, split-echo approach works with a FSE readout but would be also compatible with a GRASE imaging module [73].

In conclusion, self-calibrated, split-echo FSE is feasible for accelerated black blood imaging of the heart and of large cardiac vessels. The proposed SCSE-FSE approach is not limited to cardiovascular imaging but also meets the needs of brain, abdominal or liver imaging.
